# The C-Type Lectin OCILRP2 Costimulates EL4 T Cell Activation via the DAP12-Raf-MAP Kinase Pathway

**DOI:** 10.1371/journal.pone.0113218

**Published:** 2014-11-20

**Authors:** Qiang Lou, Wei Zhang, Guangchao Liu, Yuanfang Ma

**Affiliations:** Henan Engineering Lab of Antibody Medicine, Key Laboratory of Cellular and Molecular Immunology, Medical College of Henan University, Kaifeng 475004, China; Faculty of Medicine & Health Sciences, United Arab Emirates

## Abstract

OCILRP2 is a typical Type-II transmembrane protein that is selectively expressed in activated T lymphocytes, dendritic cells, and B cells and functions as a novel co-stimulator of T cell activation. However, the signaling pathways underlying OCILRP2 in T cell activation are still not completely understood. In this study, we found that the knockdown of OCILRP2 expression with shRNA or the blockage of its activity by an anti-OCILRP2 antagonist antibody reduced CD3/CD28-costimulated EL4 T cell viability and IL-2 production, inhibit Raf1, MAPK3, and MAPK8 activation, and impair NFAT and NF-κB transcriptional activities. Furthermore, immunoprecipitation results indicated that OCILRP2 could interact with the DAP12 protein, an adaptor containing an intracellular ITAM motif that can transduce signals to induce MAP kinase activation for T cell activation. Our data reveal that after binding with DAP12, OCILRP2 activates the Raf-MAP kinase pathways, resulting in T cell activation.

## Background

T cell activation is tightly regulated by an intricate series of signals provided by the T cell receptor/CD3 complex, cytokines, and co-stimulatory ligand/receptor systems. One of the best characterized co-stimulatory molecules expressed by T cells is CD28 [1], which interacts with CD80 (B7.1) and CD86 (B7.2) at the membrane of APCs (antigen-presenting cells). Recently, C-type lectin-like receptors (CTLRs), such as OCILRP2 [2], have emerged as a new category of T cell co-stimulatory molecules due to their ability to co-stimulate T cell proliferation and cytokine secretion. However, the signaling pathway underlying OCILRP2 is not completely understood.

Anti-CD3 or phorbol myristate acetate (PMA)-mediated MAPK activation involves the activation of Ras, leading to the activation of Raf-1 and the subsequent activation of MEK (MAPK or ERK kinase) [3]. The intracellular domain of OCILRP2 lacks the immunoreceptor tyrosine-based activation motif (ITAM) that triggers lymphocyte activation, suggesting that OCILRP2 may transmit co-stimulatory signal via adaptors, such as DAP12 [4], [5], which interacts with NKG2D (natural killer group 2, member D) in activated NK cells and CD8^+^ T cells [6]. DAP12 is a 12-kDa transmembrane protein that contains an aspartic acid residue in its transmembrane domain and a single cytoplasmic ITAM. DAP12 most likely activates SHC (Src homology 2 domain containing) transforming protein 1 via the Syk-family protein-tyrosine kinase Zap-70 [7], [8]. The sequential phosphorylation of the adaptors further triggers downstream signaling events, including the activation of the MAP and JNK kinases and nuclear translocation of transcription factors NF-AT [9], NF-κB [10], and AP-1 [11], leading to IL-2 gene expression and T cell activation. Activated T cells also produce the alpha subunit of the IL-2 receptor (CD25 or IL-2R), enabling a fully functional receptor that can bind with IL-2, which in turn activates the T cell's proliferation pathways.

OCILRP2 is a type II transmembrane CTLR that is expressed in osteoblasts, B cells, dendritic cells (DCs), and activated T cells. Splenocytes derived from OCILRP2-Ig-treated mice show a significant reduction in proliferation and level of IL-2, and the addition of OCILRP2-Ig results in a dose-dependent inhibition of CD4^+^ T cell proliferation and IL-2 production, suggesting that OCILRP2 is required for splenocyte activation [12].

The murine T cell line EL4 produces IL-2 in the presence of appropriate signals and provides a model system for analyzing T cell activation co-stimulated by H-2 and CD3 antibodies [13]. JNK phosphorylation and c-*jun* transcription were found to be induced in EL4 cells in response to phorbol ester [14]. The EL4 cell line has also been used to explore the roles of ERK activation in downstream responses. In this study, we confirmed that OCILRP2 co-stimulates T cell activation in mouse EL4 cells, and for the first time, we identify that an adaptor protein, DAP12, interacts with OCILRP2 and is involved in this T cell activation. Mechanistic studies revealed that the re-localization of OCILRP2 from the cytoplasm to the membrane under the stimulation of CD3/CD28 antibodies might be responsible for the observed T cell activation by activating the MAPK signal transduction pathway. These results provide novel insight into the mechanisms of T cell activation.

## Materials and Methods

### Cell culture

EL4 (ATCC TIB 181) cells were purchased from American Type Culture Collection and cultured as described [15]. The EL4 cells were stimulated for the indicated times with combinations of anti-CD3 (sc-18871, Santa Cruz, USA) and/or anti-CD28 antibodies (sc-12727, Santa Cruz, USA). In some experiments, an anti-IL-2 antibody (H-20, Santa Cruz, USA) or anti-OCILRP2 antibody (AF3370, R&D systems, USA) was added to the culture medium. Controls were stimulated with phorbol myristate acetate (PMA) (p1585, 50 ng/mL, Sigma, USA) and ionomycin (I3909, 100 ng/mL, Sigma, USA).

### Plasmid construction

The full-length OCILRP2 sequence was obtained from mouse B cells stimulated with anti-IgM (115-001-020, Jackson Immunotech, USA) in the presence of cycloheximide and then cloned into the pCDNA3-HA vector to yield pCDNA3-HA-OCILRP2. An OCILRP2 siRNA-expressing vector pEGFP-C3-siOCILRP2 was provided by Wenzhi Tian (Weill Medical College of Cornell University).

DAP12 was generated by PCR and introduced into the pDsRed-C1 vector or pGEX-4T-1 vector using the same method as that for OCILRP2. The full-length recombinant OCILRP2 protein GST-OCILRP2 and its two recombinant mutants, GST-OCILRP2e (N terminal region) and GST-OCILRP2i (C terminal region), were cloned into the pGEX-4T-1 cloning vector using standard molecular biology techniques. The pGEX-4T-1 recombinant vectors were then cloned into *E. coli* BL21 competent cells. GST proteins were purified over glutathione-agarose columns and stored in 50% (volume/volume) glycerol at –20°C. All constructs were sequenced and verified for accuracy, and all fusion proteins were checked for purity by SDS-PAGE and Coomassie staining.

### Cytokine IL-2 determination

EL4 cells (5×10^5^ cells/mL) were incubated with or without anti-CD3/CD28 antibodies or PMA (10 ng/mL) and ionomycin (100 ng/mL) for 12, 24, and 48 h. Supernatants were obtained by centrifugation for 3 min using a microcentrifuge. Cytokine IL-2 secreted into the supernatant was determined via ELISA (R&D systems, USA), and the samples were analyzed using a spectrophotometer (Beckman Coulter DU 640, USA). The IL-2 sample levels were determined by comparison to a standard curve of recombinant mouse IL-2 and are expressed as the mean ± S.E., as determined for each group from three independent experiments.

### Flow cytometry analysis

EL4 cells (1×10^6^ cells) were stimulated with or without anti-CD3/CD28 antibodies or PMA/ionomycin. After 48 h of stimulation, the cells were incubated with an anti-OCILRP2 antagonist antibody for 30 min on ice, followed by staining with an FITC (sc-2777)-labeled secondary antibody. The appropriate conjugated isotype-matched IgGs were used as controls. The cells were analyzed with Cellquest software using a BD FACS Calibur flow cytometer.

### T cell survival assay

The number of viable EL4 cells was monitored using Cell Counting Kit-8 (CCK-8) (C0038, Beyotime, China). To assay EL4 T cell viability with OCILRP2-mediated co-stimulation, 96-well flat-bottom microtiter plates were pre-coated with an anti-CD3/CD28 antibody, anti-CD3/CD28/OCILRP2 antibody (25 µg/mL), or anti-CD3/CD28/IL-2 antibody overnight at 4°C. The cells were also transfected with the pEGFP-siOCILRP2 plasmid and then stimulated with the anti-CD3/CD28 antibodies. 100 µL of the cell suspension (5,000 cells/well) was dispensed into each well of a 96-well plate. After incubation for 24, 48, and 72 h (37°C), 10 µL of CCK-8 solution was added to each well, and the cells were incubated for 1 h (37°C). The absorbance was measured at 450 nm using a spectrophotometer (Beckman Coulter DU 640, USA).

### GST pull-down and Co-immunoprecipitation assay

GST pull-down and co-immunoprecipitation (co-IP) were performed as described previously [16]. *E. coli* BL21 cells were harvested by centrifugation and then resuspended in buffer (PBS, 1% Triton-X 100, and 1 mM dithiothreitol (DTT)) for sonication. After centrifugation of the sonicated lysates, the supernatants were incubated with Glutathione-Sepharose 4B beads (GE) for purification. Forty-eight hours after stimulation with CD3/CD28 antibodies, EL4 cells were washed three times with ice-cold PBS and suspended in lysis buffer (20 mM Tris-HCl (pH 8.0), 200 mM NaCl, 1 mM EDTA (pH 8.0), 0.5% Nonidet P-40, 1 mM Na_3_VO_4_, 25 g/mL phenylmethylsulfonyl fluoride, 1 mM β-glycerophosphate, and 1× protease inhibitor cocktail). After shaking for 30 min (4°C), the cells were centrifuged at 12,000 x g for 10 min (4°C). The resulting supernatants were divided into three parts. One-tenth of the supernatant was boiled in 40 µl 2× SDS protein loading buffer and used for input. Equal parts of the remaining supernatant were incubated with GST or GST-fused proteins. After shaking for 2 h (4°C), the beads were washed three times with washing buffer (20 mM Tris-HCl (pH 8.0), 200 mM NaCl, 1 mM EDTA (pH 8.0), and 0.5% Nonidet P-40) and boiled in 40 µl loading buffer for SDS-PAGE.

For co-immunoprecipitation, pCDNA3-HA-OCILRP2 and pDsRed-C1-DAP12 were co-transfected into 293T cells for 48 hours. The supernatant of the co-transfected cells was immunoprecipitated with 1 µg of specific antibodies or control IgG (Santa Cruz), shaken for 2 h (4°C), mixed with 30 µL protein A/G (Santa Cruz), incubated for another 2 h (4°C), and washed three times with washing buffer. Proteins bound to the beads were boiled in 40 µL loading buffer.

### Immunofluorescence staining and Confocal microscopy

The procedure for immunofluorescence (IF) was performed as previously described [16]. Briefly, EL4 cells transfected overnight with pEGFP-C3-siOCILRP2 were stimulated with anti-CD3/CD28 antibodies. For the other groups, EL4 cells were stimulated with or without anti-CD3/CD28 or anti-CD3/CD28/OCILRP2 antibodies. All cells were incubated on glass cover slips and fixed with 3.5% paraformaldehyde for 15 min, which was then stopped by the addition of 30 mM glycine. After washing, the cells were permeabilized with 0.1% Triton X-100 for 15 min and blocked with 3% bovine serum albumin in PBS for at least 1 h (4°C). A polyclonal anti-OCILRP2 antibody (AF3370, R&D systems, USA) and monoclonal anti-DAP12 antibody (sc-133174, Santa Cruz, USA) were used at a 1∶2000 dilution. Goat anti-mouse-IgG-PE (sc-3738, Santa Cruz, USA) and rabbit anti-goat-IgG-FITC (sc-2777, Santa Cruz, USA) secondary antibodies were used at a 1∶100 dilution. Nuclei were stained with 4′, 6′-diamidino-2-phenylindole dihydrochloride (DAPI) (D9542, Sigma-Aldrich, USA), and the cells were examined by confocal microscopy (98DDFR/470111CR, Bio-Rad, USA).

### Western blot analysis

EL4 cells transfected overnight with pEGFP-C3-siOCILRP2 were stimulated with anti-CD3/CD28 antibodies. For the other two groups, EL4 cells were stimulated with or without anti-CD3/CD28 antibodies. Cell lysates were prepared in SDS sample buffer (Tris-HCl, SDS, and 20% glycerol) and detected using SDS-PAGE. The proteins were electrotransferred onto Immobilon-P PVDF membranes (0.45 µm, Millipore, USA) and probed with the appropriate primary antibodies against the following: p-Raf-1 (Ser 338) (sc-12358, Santa Cruz, 1∶200), caspase-8 p18 (G-1) (sc-166596, Santa Cruz, 1∶500), caspase-3 p17 (B-4) (sc-271028, Santa Cruz, 1∶500), JNK1 (D-6) (sc-137018, Santa Cruz, 1∶500), p-JNK1/2/3 (T183+Y185) pAb (BS4322, Bioworld Technology, 1∶500), ERK 1/2 (H-72) (sc-292838, Santa Cruz, 1∶200), p-ERK 1/2 (Thr202/Tyr204) (sc-16982, Santa Cruz, 1∶200), IκB-alpha (N-terminus) mAb (MB0106, Bioworld Technology, 1∶1000), β-actin (Sigma, 1∶5,000), and β-tubulin (Sigma, 1∶5000)). After incubation with a horseradish peroxidase-conjugated secondary antibody (Santa Cruz, 1∶10,000) for 3-4 h, the membranes were washed with PBST. Immunoreactivity was visualized using an ECL system (Perkin Elmer, USA), and densitometry scanning of the intensity of the bands was quantified using ImageJ.

### cDNA synthesis and Quantitative real-time PCR analysis

RNA from anti-CD3/CD28 antibody- or anti-CD3/CD28/OCILRP2 antibody-stimulated EL4 cells and normal EL4 cells were isolated using trizol reagent (15596-018, Invitrogen). A 3-μg of RNA was used for cDNA synthesis with a random primer mix, 10 mM dNTPs, M-MLV RT buffer, and M-MLV reverse transcriptase (Promega, Madison, USA). The RT reaction was performed at 42°C for 1 hour, followed by deactivation for 5 minutes at 90°C. cDNA for IL-2, NFκB, NFAT, MAPK8, and MAPK3 or the control household gene *β-actin* was amplified using SYBR Premix Ex Taq (RRO41A, TaKaRa, Shiga, Japan), and expression was monitored using an Rotor-gene 6000 real-time platform (Corbett Research, Australia). CT values were normalized for the expression of the *β-actin* gene.

### Statistical analysis

Experimental data were analyzed with SPSS software and compared using Student's *t*-test. Differences with a P value of <0.05 were considered statistically significant.

## Results

### Membrane translocation of OCILRP2 is involved in the co-stimulation of EL4 cell activation

**Figure 1:**
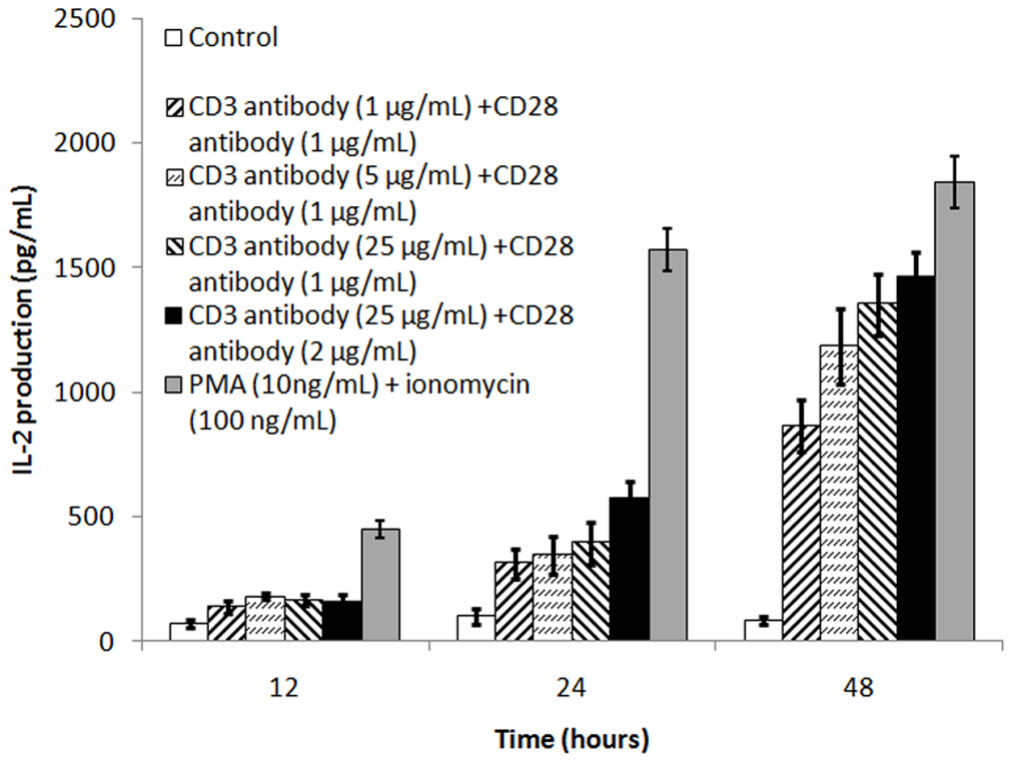
IL-2 secreted from EL4 cells after stimulation with an anti-CD3/CD28 mAb or PMA/ionomycin. EL4 cells were incubated with or without anti-CD3/CD28 antibodies (at various concentrations) or PMA (10 ng/mL) and ionomycin (100 ng/mL) for 12, 24, and 48 h. Supernatants were obtained by centrifugation for 3 min using a microcentrifuge. Cytokine IL-2 secreted into the supernatant was determined by ELISA.

It has been reported that OCILRP2 is expressed in B cells, dendritic cells (DCs), and activated T cells and is a novel co-stimulator of primary mouse T cell activation [12]. In the present study, we first investigated the effect of OCILRP2 on PMA-sensitive EL4 cell activation. EL4 cells were stimulated with PMA/ionomycin, anti-CD3/CD28 antibodies, and anti-CD3/CD28/OCILRP2 antibodies at varying concentrations pre-coated in 96-well cell plates for 12, 24, 48 h; IL-2 secretion was then analyzed by ELISA. The highest level of IL-2 secretion from EL4 cells was found after PMA/ionomycin stimulation or after combined stimulation with the anti-CD3 antibody (25 µg/mL) and anti-CD28 antibody (2 µg/mL) for 48 h (1845±103.5 pg/mL and 1464±98.55 pg/mL, respectively) (Fig. 1). This was used as the standard stimulating concentration for EL4 cell activation in the present study.

**Figure 2:**
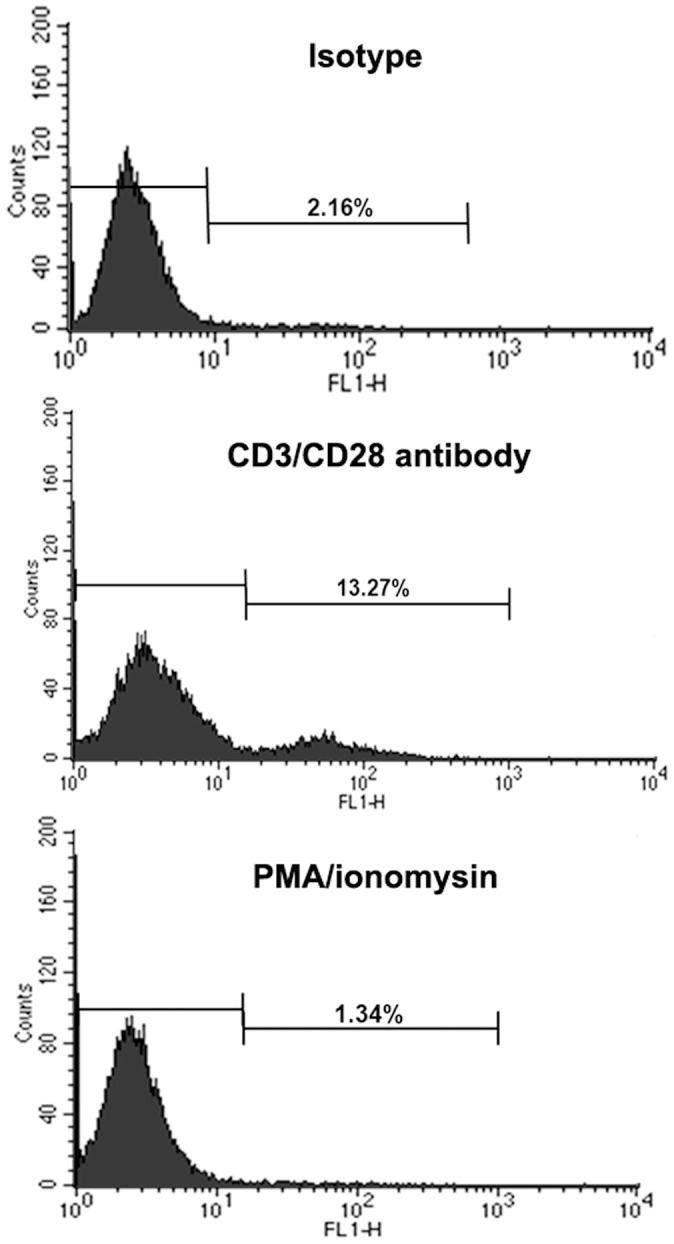
Membrane re-localization of OCILRP2 via treatment with an anti-CD3/CD28 mAb. EL4 cells were incubated with plate-bound CD3/CD28 antibodies, isotype-matched mIgGs, or PMA/ionomycin for 48 h. The cells (1×10^6^ cells) were then incubated with an anti-OCILRP2 antagonist antibody for 30 min on ice, followed by staining with an FITC-labeled second antibody. The cells were analyzed with Cellquest software using a FACS Calibur flow cytometer.

After the stimulation of EL4 cells by CD3/CD28 antibodies, most of the OCILRP2 proteins are transferred from the cytoplasm to the cell membrane, which was confirmed by a flow cytometric analysis. Although 2.16% of the ‘resting’ EL4 cells expressed OCILRP2, the percentage of EL4 cells expressing OCILRP2 increased to 13.27% at 48 h following CD3/CD28 stimulation (Fig. 2). However, the PMA/ionomycin-treated EL4 cells did not exhibit this membrane translocation.

### An antagonist OCILRP2 antibody reduces the viability of EL4 cells stimulated by CD3/CD28 antibodies

**Figure 3:**
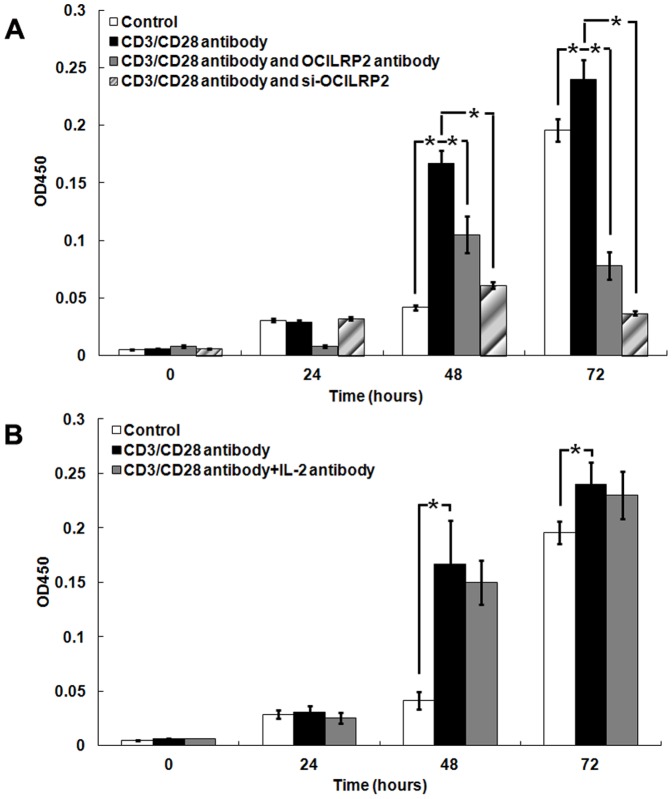
OCILRP2 co-stimulation affects EL4 cell viability. Six-well flat-bottom microtiter plates were pre-coated with anti-CD3/CD28 antibodies or anti-CD3/CD28/OCILRP2 antibodies (a) or anti-CD3/CD28/IL-2 antibodies (b) overnight at 4°C. Cells were transfected with pEGFP-siOCILRP2 plasmid and then stimulated with the anti-CD3/CD28 antibodies (a). Then, 100 µL of the cell suspension (5,000 cells/well) was dispensed into each well of a 96-well plate. After incubation for 24, 48, and 72 h (37°C), 10 µL of CCK-8 solution was added to each well, and the cells were incubated for 1 h (37°C). The absorbance was measured at 450 nm using a spectrophotometer (Beckman Coulter DU 640, USA). *P<0.05.

Because OCILRP2 functions in EL4 cell activation, the role of OCILRP2 in EL4 cell viability was further examined using a CCK8 cell proliferation kit. Incubation with the anti-CD3/CD28 antibody for 48 h or 72 h promoted viability. However, the simultaneous incubation with the anti-OCILRP2 antagonist antibody or the knock-down of OCILRP2 expression significantly reduced viability by approximately 1.59 and 2.73 times at 48 h and 3.08 and 6.54 times at 72 h, respectively (Fig. 3a). However, the cell cycle percentages did not change notably (Fig. S1).

To determine whether the observed EL4 cell viability was caused by the direct effect of OCILRP2 or the high level of IL-2 secreted by the cells, the extent of EL4 cell viability with anti-CD3/CD28/IL-2 or anti-CD3/CD28 antibody stimulation were compared. Similar cell viability was observed (Fig. 3b).

### OCILRP2 interacts with DAP12 in vitro and in vivo

**Figure 4:**
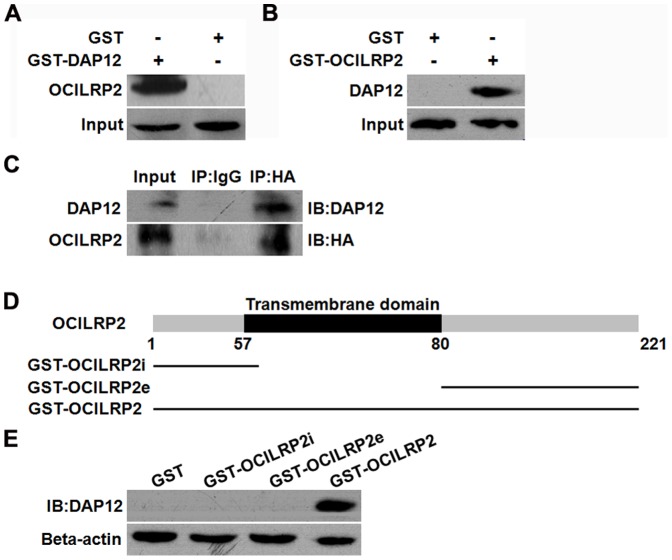
Interaction of OCILRP2 and DAP12 in GST pull-down and co-immunoprecipitation assays. The GST pull-down assay was carried out using purified beads that contained GST, GST-DAP12, or GST-OCILRP2. Precipitated OCILRP2 or DAP12 was detected by western blotting using an anti-OCILRP2 or anti-DAP12 antibody, respectively (a, b). 293T cells were grown in 6-cm dishes and transfected with the pCDNA3-HA-OCILRP2 and pDsRed-C1-DAP12 plasmids, respectively. OCILRP2 and DAP12 were detected by western blotting using an anti-HA antibody or an anti-DAP12 antibody (c). Schematic diagram of OCILRP2 predicted by SMART software. The green column represents the transmembrane region (amino acids 57–80) (d). The GST pull-down assay was carried out using purified beads that contained GST, full-length GST-OCILRP2, GST-OCILRP2i (aa 1–57), or GST-OCILRP2e (aa 80–221). Precipitated DAP12 was detected by western blotting using an anti-DAP12 antibody (e).

Because DAP12 contains an immunoreceptor tyrosine-based activation motif (ITAM) region that transmits the activation signal, we then investigated the contribution of DAP12 to membrane translocation. To determine the mechanism of the effect of OCILRP2 on T cell activation, we assessed OCILRP2 interactions with the adaptor protein DAP12, as the intracellular region of OCILRP2 is relatively short and may be insufficient to transmit a signal. A GST pull-down assay was performed to verify the OCILRP2/DAP12 interaction. GST-fused OCILRP2 was expressed in *E. coli* (BL21/DE3) and purified on glutathione-Sepharose 4B beads. After incubation with EL4 cell lysates, the precipitated proteins were separated by SDS-PAGE and detected by western blotting using an anti-DAP12 antibody. The results showed that DAP12 bound to GST-OCILRP2 but not to GST alone (Fig. 4a). A reciprocal experiment using GST-DAP12 and OCILRP2 gave a similar result (Fig. 4b). To show that OCILRP2 also interacts with DAP12 in cells, 293T cells were co-transfected with the pCDNA3-HA-OCILRP2 and pDsRed-C1-DAP12 plasmids, and interaction between OCILRP2 and DAP12 was observed by co-immunoprecipitation with an anti-HA or anti-DAP12 antibody (Fig. 4c).

To better understand the function of the association between OCILRP2 and DAP12, it was necessary to map the amino acid sequences required for their binding. Thus, a protein truncation assay was performed to identify the region of OCILRP2 that interacts with DAP12. Because OCILRP2 has a transmembrane region and a C-type lectin-like cytoplasmic domain structure (predicted by SMART software), OCILRP2 was truncated at its N-terminal (aa 1–57) and C-terminal (aa 80–221) regions based on a prediction model (Fig. 4d) and fused with GST. The GST pull-down results showed that only the full-length, but not the N-terminal region or C-terminal region, of OCILRP2 bound to DAP12 (Fig. 4e), indicating that the interaction of OCILRP2 with DAP12 maps to the transmembrane region: aa 58–79.

**Figure 5:**
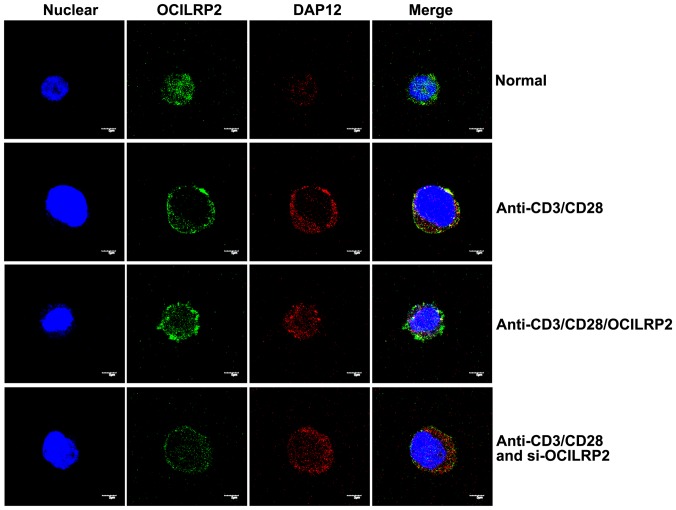
Membrane co-localization of OCILRP2 and DAP12 under anti-CD3/CD28 mAb treatment in EL4 T cells. EL4 T cells pre-transfected with pEGFP-siOCILRP2 or pre-coated with the OCILRP2 Ab were cultured overnight in the presence or absence of an anti-CD3/CD28 mAb and then stained for OCILRP2 (green) and nuclear stained with DAPI (blue) and DAP12 (red) to study OCILRP2 and DAP12 protein expression and localization. Unstimulated EL4 T cells exhibited OCILRP2 protein expression in the cytoplasm (upper panels). In contrast, CD3/CD28-activated EL4 T cells showed intracellular and membrane OCILRP2. OCILRP2/DAP12 co-localization appears in yellow. Each picture is representative of the vast majority of the observed cells on the slides.

To investigate in detail the mechanism responsible for T cell activation upon OCILRP2 stimulation, we tested the OCILRP2/DAP12 interaction in EL4 cells by confocal laser microscopy (CLSM) after immunological staining with PE- or FITC-labeled antibodies. EL4 cells were stimulated with CD3/CD28 antibodies, CD3/CD28/OCILRP2 antibodies, or CD3/CD28 antibodies and then transfected with an OCILRP2-interfering plasmid. Our data showed a cell membrane colocalization of OCILRP2 with DAP12 (Fig. 5). OCILRP2 trafficked from the cell cytoplasm to the cell membrane, and strong merge signals were observed in the cell membrane regions in EL4 cells.

### OCILRP2 participates in anti-CD3/CD28 antibody-induced EL4 cell activation by promoting Erk and Jnk phosphorylation

**Figure 6:**
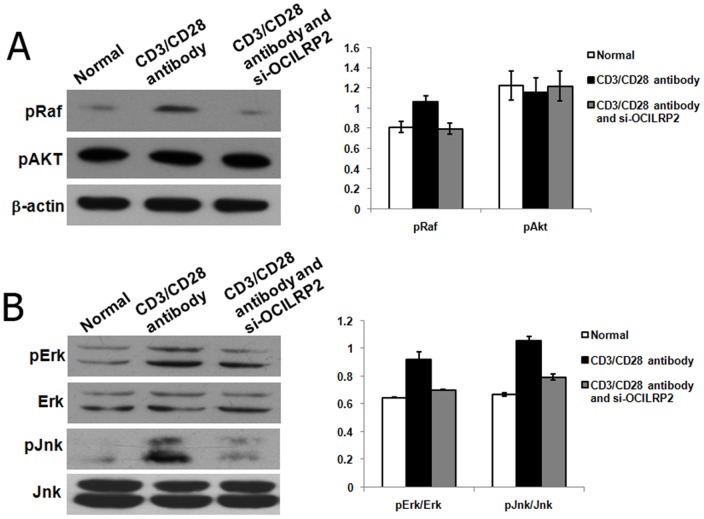
Western blot analysis of Raf, Erk, Jnk, and Akt phosphorylation in EL4 cells. EL4 T cells were incubated in DMEM medium with an immobilized anti-CD3/CD28 mAb in the presence or absence of si-OCILRP2. After incubation at 37°C for 48 h, the T cells were lysed, and aliquots of 20 µg of the whole-cell lysate were analyzed by western blotting using antibodies against phospho-Erk, Erk, phospho-Jnk, and Jnk (a) and phospho-Raf and phosphor-Akt (b). The data are expressed as a percentage of the level of active Erk or Jnk of the total Erk or Jnk. Each data point represents the mean ± S.E. from three separate experiments.

Because OCILRP2 was demonstrated to participate in anti-CD3/CD28 antibody-induced activation as well as in the secretion of IL-2 by EL4 cells, the effects of anti-CD3/CD28 antibodies and si-OCILRP2 on the expressions of the MAPK and PI3K/Akt pathways were investigated by western blotting to clarify the underlying mechanisms. The results of western blotting showed that anti-CD3/CD28 antibodies induced the phosphorylation of ERK1/2 and JNK (Fig. 6a). In contrast, knock-down of OCILRP2 inhibited the anti-CD3/CD28 antibody-induced phosphorylation of ERK1/2 and JNK in EL4 cells. However, anti-CD3/CD28 antibodies did not significantly affect the phosphorylation of Akt (Fig. 6b).

Because ERK1 and JNK are involved in EL4 cell IL-2 expression, Raf was examined to determine whether it is involved in this signaling pathway. The results showed that the phosphorylation of Raf was inhibited by si-OCILRP2 (Fig. 6b).

### An anti-OCILRP2 antagonist antibody decreased the transcriptional levels of NF-κB, NFAT, MAPK3, MAPK8, and IL-2

**Figure 7:**
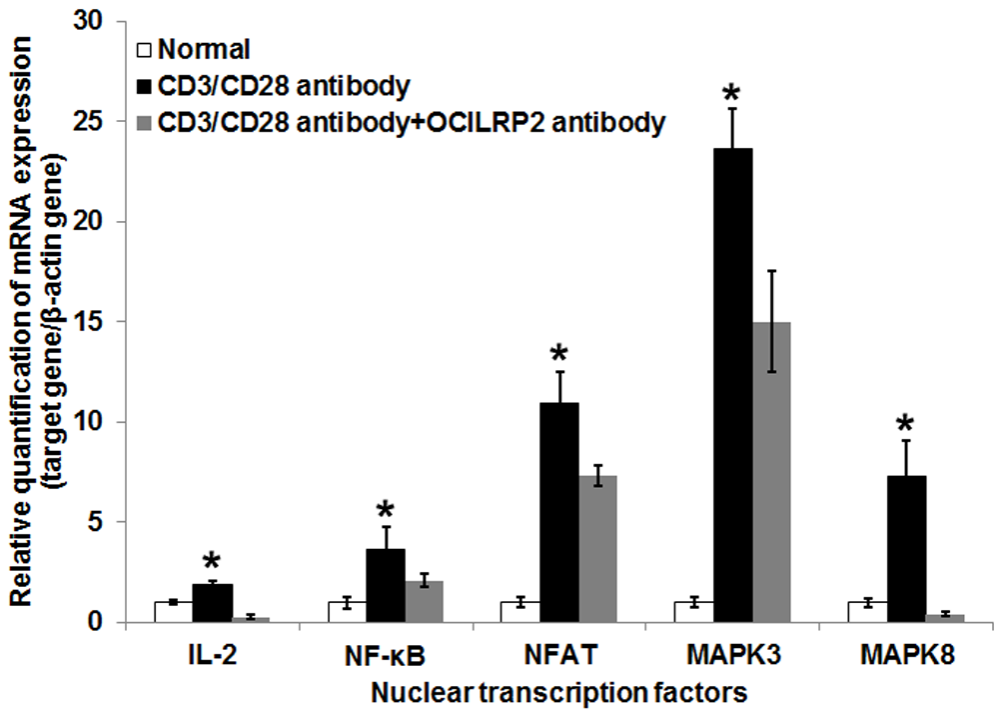
mRNA expression in EL4 cells stimulated with or without anti-CD3/CD28 antibodies or anti-CD3/CD28/OCILRP2 antibodies. RNA from un-stimulated or CD3/CD28 antibody- or CD3/CD28/OCILRP2 antibody-stimulated EL4 cells was isolated using the RNeasy kit (Qiagen). cDNA of IL-2, NF-κB, NFAT, MAPK3, and MAPK8 or the control household gene *β-actin* was amplified using SYBR Premix Ex Taq (RRO41A, TaKaRa, Shiga, Japan), and expression was monitored using an Rotor-gene 6000 real-time platform (Corbett Research, Australia). Ct values were normalized for the expression of the *β-actin* gene. *P<0.05.

The NF-κB and NFAT (nuclear factor of activated T cells) proteins are key regulators of T cell development and function [17]. Changes in the levels of NF-κB and NFAT transcription were compared after stimulation by CD3/CD28 antibodies with or without the anti-OCILRP2 antagonist antibody. NF-κB and NFAT transcripts were preferentially expressed (up-regulated 3.6 and 10.9 times, respectively) in the EL4 cells stimulated with CD3/CD28 antibodies. Additionally, after CD3/CD28 antibody stimulation, the expression of Interleukin-2 (IL-2), MAPK3 (coding for ERK1/2), and MAPK8 (coding for JNK1) were also increased by approximately 1.9, 23.6, and 7.3 times (P<0.05), respectively (Fig. 7).

## Discussion

In the present study, the costimulation of mouse T cell activation by the C-type lectin-like molecule OCILRP2 was confirmed in the EL4 cell line. In addition, OCILRP2 redistribution to the cell membrane and its interaction with the adaptor protein DAP12 was likely the cause of EL4 cell activation.

Wenzhi Tian et al. [12] first reported the effects of OCILRP2 on primary human T cell proliferation and IL-2 production. These authors found that silencing OCILRP2 leads to intrinsic defects in T cell survival as well as cell cycle progression in response to TCR and CD28 signaling. Recently, further studies on the possible mechanism of T cell activation have been reported. Thebault P et al. demonstrated that the C-type lectin-like receptor CLEC-1, expressed by myeloid cells and endothelial cells, is up-regulated by immunoregulatory mediators and moderates T cell activation [18]. Another study by Clifford S. Guy et al. reported that T cell proliferation requires the recruitment of Vav1 to the CD3 complex and activation of the Notch signaling pathway [19]. However, due to the lack of signaling motifs in its intracellular domain, the mechanism underlying OCILRP2-mediated T cell co-stimulation has remained undefined.

OCILRP2 lacks cytoplasmic signaling motifs but contains charged residues in its transmembrane domains, which may allow associations with signaling partners, such as homodimers DAP12 or DAP10 (Fig. S2). The 2 homodimers initiate distinct signaling cascades: DAP12 activates the Syk/ZAP70 pathway, and DAP10 signals through the PI3K pathway [20]–[24]. Western blot analyses with antibodies against phosphorylated Akt revealed equal protein levels in EL4 cells treated with CD3/CD28 antibodies or CD3/CD28/OCILRP2 antibodies, thus demonstrating that the co-stimulatory activation of OCILRP2 may not occur via the DAP10 signaling pathway. DAP12 homodimers associate with a variety of receptors expressed by macrophages, monocytes and myeloid cells including TREM2 [25], Siglec H [26] and SIRP-beta [27], as well as activating KIR [28] and the NKG2C proteins [29] expressed by NK cells. In this study, an interaction between OCILRP2 and DAP12 was demonstrated, and the interactions can be mediated by transmembrane domain of OCILRP2. OCILRP2 has a short cytoplasmic tail (57 amino acids) that lacks an ITAM or other tyrosine motif, may recruit adaptor protein to activate downstream signaling pathways during T cell activation. DAP12 may be playing a role in enhancing the maturation and stabilization of OCILRP2 or that DAP12 has a role in impacting the trafficking of OCILRP2 to the cell surface. In NK cells, spleen tyrosine kinase (SYK) and ζ -chain-associated protein kinase of 70 kDa (ZAP70) are recruited to the plasma membrane following DAP12 phosphorylation, leading to the activation of phosphatidylinositol 3-kinase (PI3K) and tyrosine phosphorylation of the scaffolding proteins LAT (linker for activation of T cells) and LcK (Leukocyte C-terminal Src kinase) [30]. Interestingly, Wenzhi Tian et al. [2] recently identified reduced LcK tyrosine phosphorylation in OCILRP2-silenced T cells, whereas the tyrosine phosphorylation of LAT was unchanged, suggesting that DAP12 may transmit the OCILRP2 co-stimulatory signal via the DAP12-Lck pathway.

Our laboratory has utilized EL4, a murine thymoma cell line, as a model system in which to explore the intracellular signaling pathways that are activated upon ligation of the co-stimulatory receptor OCILRP2. The anti-CD3 antibody-induced activation of EL4 cells was enhanced in the presence of an immunologically cross-linked and immobilized anti-H2 antibody [13]. In the present study, our results demonstrate that OCILRP2 does not cooperate with either anti-CD3 or -CD28 antibodies to enhance IL-2 release; rather, the effect of OCILRP2 was observed only when the cells were stimulated with a combination of both antibodies. Therefore, OCILRP2 overexpression or translocation does not replace signaling by either of the two receptor and does not appear to be uniquely coupled to TCR or CD28. In addition, an anti-IL-2 antibody did not affect OCILRP2-mediated viability, suggesting that IL-2 is not the primary stimulus driving the expansion of anti-CD3 plus anti-CD28-stimulated T cells.

The mechanism by which OCILRP2 co-stimulation enhances TCR signals is not clear. CD28 enhances TCR signaling by stimulating lipid raft redistribution at the site of TCR engagement [31]. Moreover, there is evidence demonstrating that CD28 can translocate to lipid rafts upon cross-linking and that this correlates with its co-stimulation of IL-2 production [32]. OCILRP2 may play a role similar to that of CD28 by facilitating TCR-mediated raft microdomain formation. T cell receptor signal transduction is initiated by the assembly and aggregation of signaling complexes including the adaptor molecule LAT [33], the Src homology 2 domain-containing leukocyte protein of 76 kDa (SLP-76) [34], Lck [35], and Fyn [36]. OCILRP2 may be recruited to lipid rafts and facilitate Lck tyrosine phosphorylation. Because human DAP12 reportedly binds the SH2 domains of Syk and Zap-70 [7], the ligation of OCILRP2 may lead to the activation of Syk and Zap-70.

MAPKs are regarded as key switches of cellular activation and proliferation [37]. Within the context of co-stimulatory OCILRP2 signaling, an increase in Jnk or Erk phosphorylation was observed. The phosphorylation of Raf was inhibited by si-OCILRP2, suggesting that Raf might be essential for the regulation of CD3/CD28 antibody-induced IL-2 expression.

The pathway revealed by our findings provides a molecular basis for the defective TCR-mediated activation of OCILRP2-silenced mouse T lymphocytes (Fig. S3). Therefore, selective pharmacological strategies designed to modulate the recruitment of OCILRP2 to the cell membrane may prove therapeutically useful for modulating T cell co-stimulatory signals in immunological diseases.

## Conclusions

In the present study of anti-CD3/CD28 antibodies, OCILRP2 re-localized to the EL4 T cell membrane and interacted with the adaptor protein DAP12 to transmit the T cell activation signal, leading to the activation of the downstream MAPK signaling pathway and nuclear transcription factors in the murine EL4 T cell line.

## Supporting Information

Figure S1 **Analysis of cell cycle progression.** EL4 cells were untreated or treated with anti-CD3/CD28 antibodies, an anti-OCILRP2 antagonist antibody, or si-OCILRP2. The cells were harvested after 48 h, fixed, stained, and analyzed for DNA content. The distribution and percentage of cells in pre-phase and G1, S, and G2/M phases of the cell cycle are indicated.
(TIF)

Figure S2 **Postulated schematic diagram of OCILRP2 and DAP12 interaction.**
(TIF)

Figure S3 **Schematic model for the mechanism of OCILRP2-stimulated T cell activation.**
(TIF)
