# Supplementary figures and images for: The C-Type Lectin OCILRP2 Costimulates EL4 T Cell Activation via the DAP12-Raf-MAP Kinase Pathway
Source: PLoS One. 2014 Nov 20;9(11):e113218. doi: 10.1371/journal.pone.0113218 (PMC4239057; doi:10.1371/journal.pone.0113218)

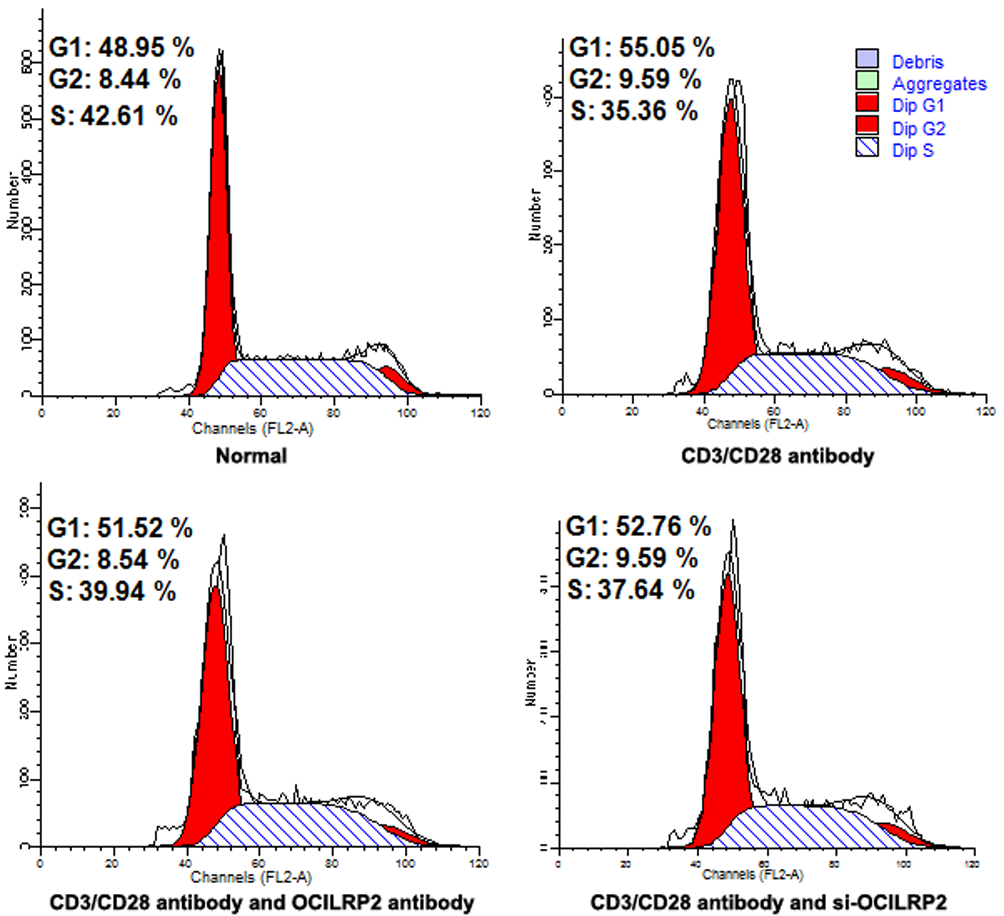

Supplement: Figure S1 — Analysis of cell cycle progression. EL4 cells were untreated or treated with anti-CD3/CD28 antibodies, an anti-OCILRP2 antagonist antibody, or si-OCILRP2. The cells were harvested after 48 h, fixed, stained, and analyzed for DNA content. The distribution and percentage of cells in pre-phase and G1, S, and G2/M phases of the cell cycle are indicated. (TIF) [file pone.0113218.s001.tif]

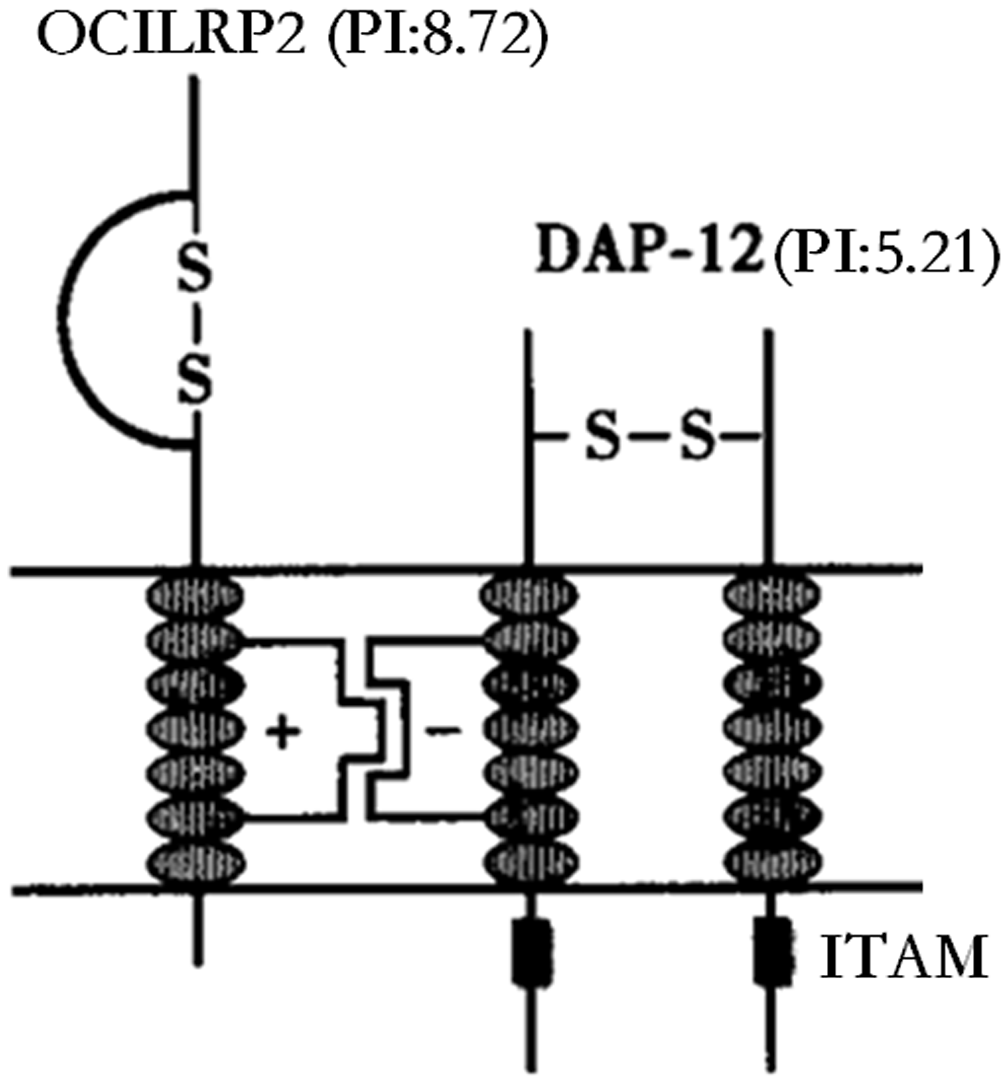

Supplement: Figure S2 — Postulated schematic diagram of OCILRP2 and DAP12 interaction. (TIF) [file pone.0113218.s002.tif]

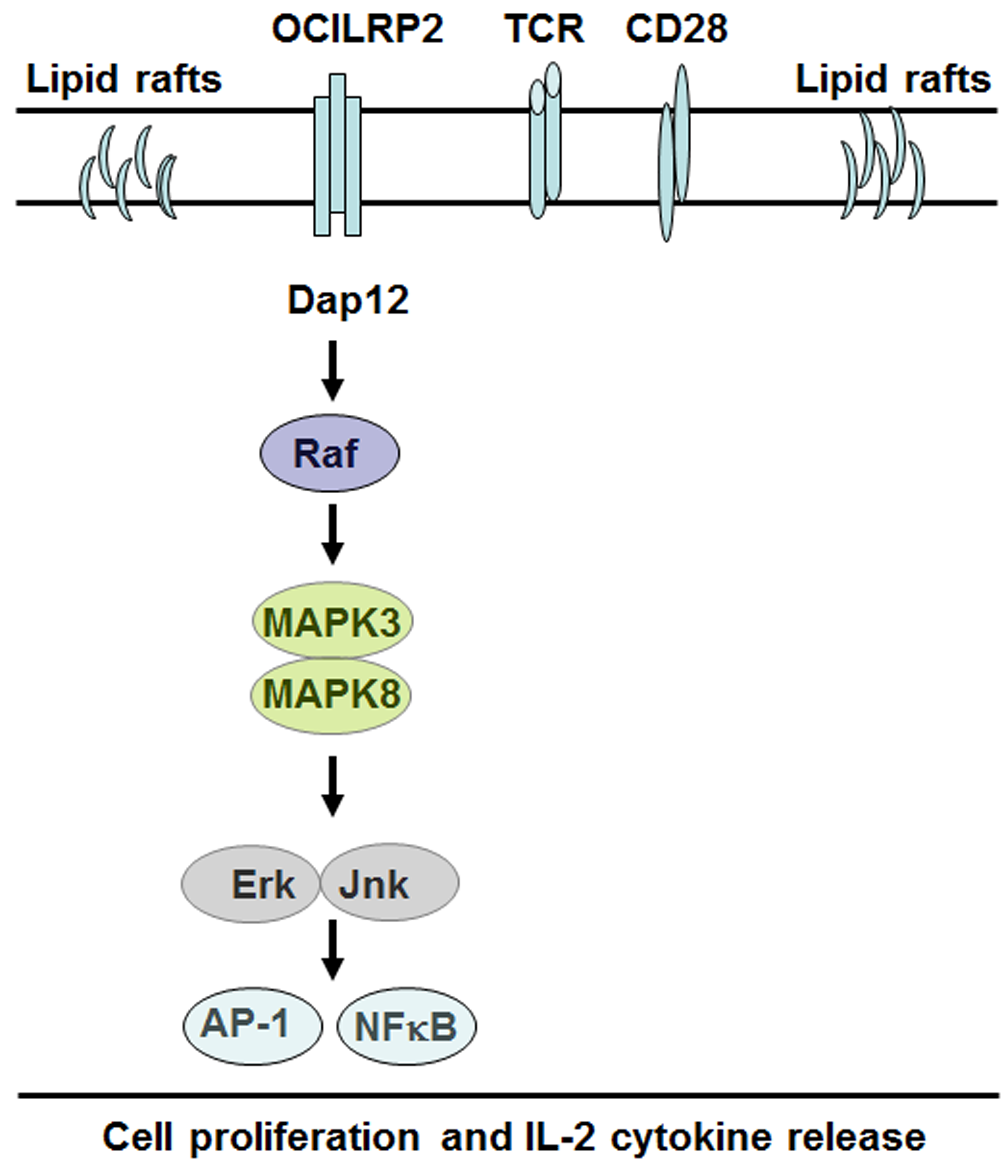

Supplement: Figure S3 — Schematic model for the mechanism of OCILRP2-stimulated T cell activation. (TIF) [file pone.0113218.s003.tif]
